# Fundamental and realized feeding niche breadths of sexual and asexual stick insects

**DOI:** 10.1098/rspb.2018.1805

**Published:** 2018-11-28

**Authors:** Chloé Larose, Darren J. Parker, Tanja Schwander

**Affiliations:** 1 Department of Ecology and Evolution, University of Lausanne, Quartier Unil-Sorge, CH-1015 Lausanne, Switzerland; 2 Swiss Institute of Bioinformatics, Lausanne, Switzerland

**Keywords:** degree of specialization, herbivorous insect, host plant range, realized versus fundamental niche, sexual versus asexual reproduction, *Timema* stick insects

## Abstract

The factors contributing to the maintenance of sex over asexuality in natural populations remain unclear. Ecological divergences between sexual and asexual lineages could help to maintain reproductive polymorphisms, at least transiently, but the consequences of asexuality for the evolution of ecological niches are unknown. Here, we investigated how niche breadths change in transitions from sexual reproduction to asexuality. We used host plant ranges as a proxy to compare the realized feeding niche breadths of five independently derived asexual *Timema* stick insect species and their sexual relatives at both the species and population levels. Asexual species had systematically narrower realized niches than sexual species, though this pattern was not apparent at the population level. To investigate how the narrower realized niches of asexual species arise, we performed feeding experiments to estimate fundamental niche breadths but found no systematic differences between reproductive modes. The narrow realized niches found in asexual species are therefore probably a consequence of biotic interactions such as predation or competition, that constrain realized niche size in asexuals more strongly than in sexuals.

## 1. Introduction

The maintenance of obligate sex in natural populations, despite numerous disadvantages compared to other reproductive systems, is a major evolutionary paradox. Although there is a rich body of theory proposing potential benefits of sex, empirical studies evaluating such benefits under natural conditions remain scarce [1]. A simple mechanism that could contribute to the maintenance of reproductive polymorphisms is niche differentiation between sexual and asexual species [2–7]. Such niche differentiation could result from a difference in ecological optima between sexuals and asexuals [3–5], or from situations where sexual species cover larger fractions of the available niche space than their asexual counterparts [6].

**Figure 1.**
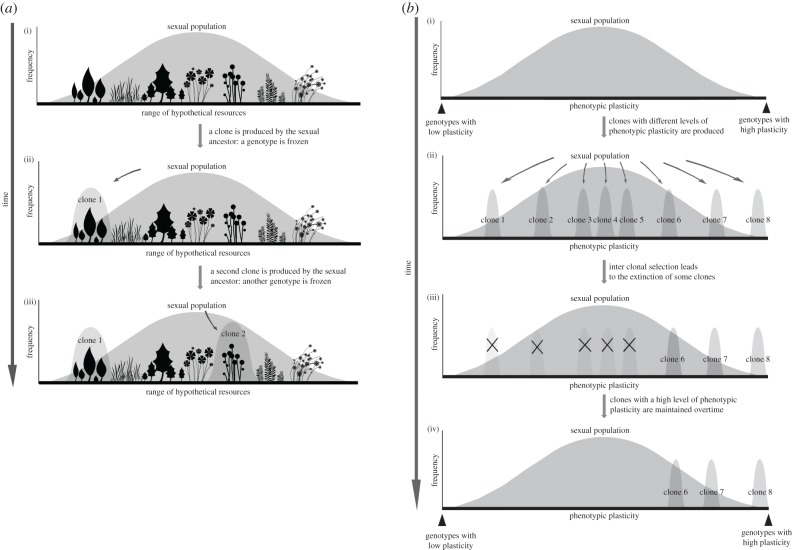
(*a*) The frozen niche variation model. (i) A sexual population (broad curve) exhibits genetic variation for the niche use (here symbolized by a range of hypothetical plants); (ii) a new asexual clone is produced, comprising a subset of the genotypic diversity contained in its sexual ancestor; (iii) a second clone is produced from a different sexual genotype characterized by a different ecological niche. The niche breadth of the sexual population as a whole is larger than the one of each individual clone. Adapted from Vrijenhoek & Parker [11]. (*b*) General-purpose genotype. (i) Individuals in a sexual population vary in the range of their environmental tolerances (narrow to broad plasticity); (ii) clones are produced from different genotypes in the sexual population with different levels of plasticity; (iii,iv) natural selection favours clones with broader tolerances such that clones may feature higher levels of plasticity than the sexual population as a whole (e.g. extreme case of clone 5). Figure adapted from Vrijenhoek & Parker [11].

Because asexual species derive from sexual ancestors, fundamental niches (i.e. the range of environmental conditions that allow for survival, growth and reproduction) in new asexual species should depend directly upon the fundamental niche found in the ancestral sexual species. However, how the fundamental niche in an ancestral sexual population translates to that found in an asexual population is unclear. For example, the *frozen niche variation* (FNV) model predicts that the phenotypic distribution of a new, recently derived asexual would be narrower than that of its genetically variable sexual ancestor, because a single sexual genotype will be ‘frozen’ in the new asexual lineage [3,8–10]. This may result in different fundamental niche breadths between sexual and asexual species, with the new asexual species being more specialized and able to exploit fewer niches than the sexual species from which it is derived from ([8,11]; figure 1*a*). By contrast, the ‘*general-purpose genotype*’ (GPG) hypothesis [12–14] proposes that asexual lineages should generally have broader environmental tolerances than sexual individuals because of strong selection for plasticity in asexuals. Indeed, a temporally and spatially variable environment should favour, among all the independently derived asexual clones, those with the broadest environmental tolerances. Under this scenario, we would expect asexual populations to have broader ecological niches than sexual ones (figure 1*b*). The two hypotheses are non-mutually exclusive. For example, the FNV model can be applied to sexual genotypes with different levels of plasticity—a specific plasticity level will be ‘frozen’ in the new asexual lineage, depending on the sexual genotype it derives from [11]. Furthermore, by combining the FNV and GPG, we can suggest that young asexual lineages would feature, on average, narrow niches, while old ones would feature broad niches.

Regarding the breadth of the realized niche (i.e. the fraction of the fundamental niche used by organisms under natural conditions), there is currently no specific theory predicting similarities or differences between sexuals and asexuals. There are, however, several theories predicting that sex can accelerate the rate of adaptation compared to asexuality [15–18]. Sexual organisms therefore may be able to evolve adaptations to competitors, pathogens or predators more rapidly than asexuals. As a consequence, the realized niche in asexual organisms may be smaller than in sexual organisms owing to a reduced ability to respond to these biotic pressures.

**Figure 2.**
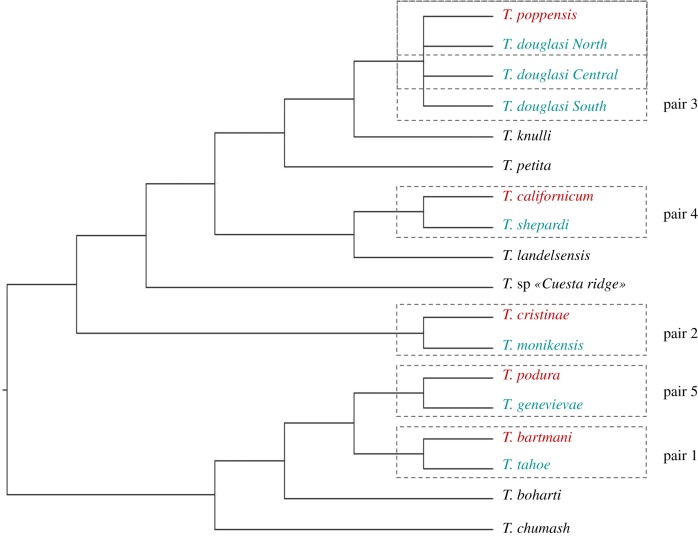
*Timema* species phylogeny. Phylogeny redrawn from Riesch *et al.* [20] with the seven asexual lineages added from Schwander *et al.* [19] (in blue). The used sexual species are labelled in red. Pair numbers correspond to the most recent (i.e. pair 1) to the most ancient (i.e. pair 5) transition to asexuality (ranking from Bast *et al.* [21]).

Here, we evaluate whether asexuality is associated with different niches and niche sizes than sexual reproduction, using herbivorous stick insects of the genus *Timema* as a model system and different host plants as a proxy for different niches. Seven independently derived asexual lineages have been identified in this genus, each with a closely related sexual counterpart ([19]; figure 2). This allows us to perform replicate comparisons between sexual and asexual lineages. Moreover, the asexual *Timema* lineages vary in age [19,22], allowing us to assess the possible consequences of asexuality on niche breadth over a range from recently derived to long-term asexuality.

We first estimated the size of the realized feeding niches of sexuals and asexuals both at the species and at the population level in five sexual–asexual *Timema* sister species pairs, using occurrences on different host plants in natural populations. *Timema* feed on the leaves or the needles of very diverse host plants, comprising both angiosperms and conifers, and the quality of these plants as a food source is highly variable [23]. We then conducted feeding experiments with species from four sexual–asexual species pairs to estimate the size of their fundamental feeding niches. Finally, we evaluated the contribution of predation to shaping realized niches in sexuals and asexuals. *Timema* are characterized by different cryptic morphs on different host plants, both within and between species [24–26]. Previous studies have shown that the combination of selection imposed by predators and *Timema* host preference maintain a correlation between morph frequency and host plant frequency between populations [24,27,28], indicating that colour polymorphism and predation may be of key importance for realized niches in *Timema*.

## 2. Material and methods

### (a) Realized feeding niche breadths

Different host plants are generally considered to reflect different ecological niches for herbivorous insects [29] and we therefore chose to study the host plants used by *Timema* under natural conditions as a proxy for their realized niches.

Data from a previous study that collected information on host plant use across all 23 known *Timema* species [23] allowed us to estimate the size of the realized feeding niche of sexuals and asexuals at the species level. To estimate the realized niche at the population level, we further performed a count of the number of individuals collected on each potential host plant across 30 populations from five species pairs (between two and six populations per species; electronic supplementary material, table S2). The size of the realized feeding niche per population was then quantified with the inversed Tau (*τ*) specialization index [30], which ranges from 0 (pure specialist) to 1 (complete generalist), calculated as follows:
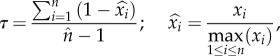
where *n* corresponds to the number of *Timema* host plants found in a given location, *x_i_* represents the frequency of occurrence on plant *i* and max (*x_i_*) is the highest frequency of occurrence for the focal population in this location.

### (b) Degree of colour polymorphism

Realized feeding niches depend on multiple factors, including the fundamental niches as well as species interactions (notably predation and competition). *Timema* are characterized by different cryptic colour morphs and previous studies have shown that selection imposed by predators favours different colour morph sets on different plants [24,28,31]. To develop insights into the contribution of predators to the sizes of the realized feeding niches in *Timema*, we thus quantified colour polymorphism at the species and population levels.

Colour phenotypes vary broadly in several *Timema* species but can be separated into a total of 14 discrete morphs across all species (range 1–8 per species; electronic supplementary material, table S1). We recorded colour morph frequencies from all sampling locations (electronic supplementary material, table S2) and used the Simpson diversity index to quantify the level of polymorphism [32]. This index varies between 0 (here indicating colour monomorphism) and 1 (indicating the diversity of equally frequent colour morphs). We then estimated the correlation between the degree of colour polymorphism and the size of the realized feeding niche, both at the species and at the population levels with phylogenetic generalized least squares (PGLS) to account for phylogenetic non-independence among *Timema* species. These analyses were conducted using the ape [33] and nlme [34] R packages [35] using a Brownian motion model for trait evolution.

### (c) Fundamental feeding niche breadths

To estimate the fundamental feeding niche breadths of sexual and asexual *Timema* species, we performed a feeding experiment and measured insect performance on different host plants. We chose seven plants known to be commonly used by several *Timema* species while trying to cover the phylogenetic diversity of the host plants [23]. Specifically, we chose four angiosperms: (*Ceanothus thyrsiflorus* (lilac, lil), *Adenostoma fasciculatum* (chamise, cha), *Quercus agrifolia* (oak) and *Arctostaphylos glauca* (manzanita, mz)), and three conifers: (*Pseudotsuga menziesii* (douglas fir, df), *Abies concolor* (white fir, wf) and *Sequoia sempervirens* (redwood, rdw)). Stick insects from eight *Timema* species (four sexual–asexual species pairs) were collected from multiple field sites in California (electronic supplementary material, table S3). We only used fourth-instar juvenile females for feeding experiments to minimize age-related effects on insect performance during our experiments. Between 10 and 20 such females were used per host plant for a total of 70–105 females per population (635 insects in total; electronic supplementary material, table S3). The females were installed individually in tubes closed with a net, each containing a fresh branch from one of the seven plants of the experiment, as described in [23], to measure survival and weight gain after 10 days.

We first used a generalized linear model (GLM) with a binomial error to compare survival and an ANOVA to compare the weight gain of all stick insects species on the different plants using R [35]. We then compared for each *Timema* species pair separately, the survival and weight gain of the sexual and asexual individuals, testing specifically for an interaction between reproductive mode and plant species because a significant interaction between these two factors would indicate a difference in fundamental feeding niche between sexuals and asexuals. Finally, we estimated the breadth of the fundamental feeding niche of the eight *Timema* species using again the inversed Tau index (calculated as described above; but where *x_i_* represents the survival or weight gain on plant *i*, and max (*x_i_*) represents the best survival or weight gain for a given *Timema* species). We could not compare the fundamental feeding niche of the *Timema bartmani*/*Timema tahoe* species pair because *T. tahoe* individuals of the appropriate developmental stage could not be collected in sufficient numbers for the feeding experiment.

## 3. Results

**Figure 3.**
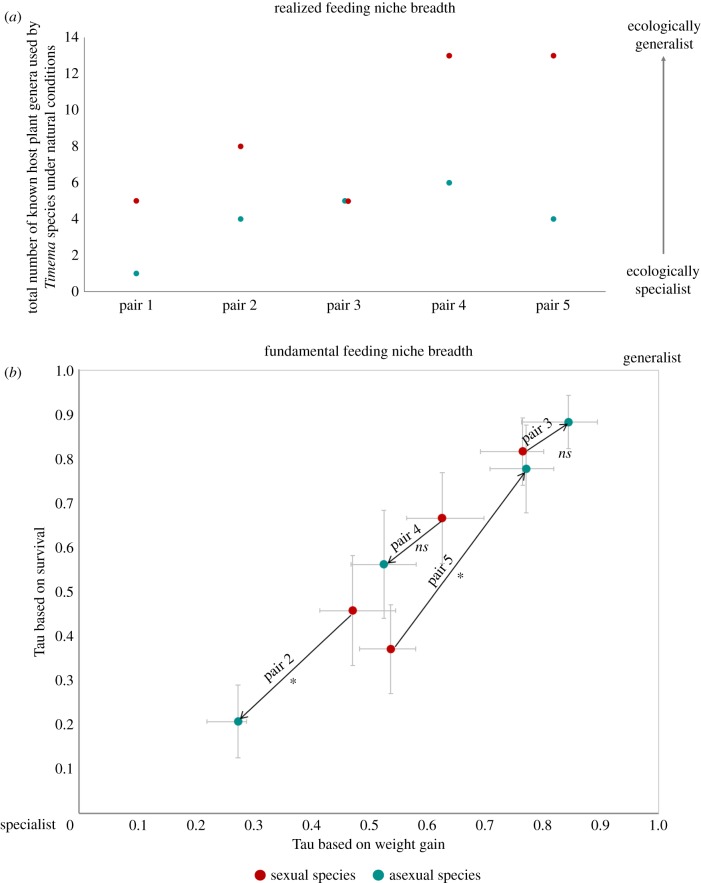
Realized (*a*) and fundamental (*b*) feeding niche breadths of sexual and asexual *Timema* stick insects. The pairs are listed from the most recent to the most ancient transition to asexuality (ranking from Bast *et al.* [21]). Stars indicate significant differences of the Tau indices of the sexual and asexual species of a pair. For species pair numbers, see figure 2.

For realized niches measured at the species level, the sexuals are more ecologically generalist in four out of five cases, as they used at least twice as many different plant genera as their asexual relatives (figure 3*a*). In the remaining case (*Timema poppensis/Timema douglasi*), the sexual and the asexual species used the same number of host plants in the wild (figure 3*a*). For realized niches measured at the population level, all 10 species are specialized, feeding typically on one or two host plants species even when additional species are available (Tau indices varying between 0 and 0.48; electronic supplementary material, figure S1B). There were no significant differences in the degree of specialization between sexual and asexual populations (GLM; *p*-value = 0.19). However, we did find that (within species) sexual populations vary more than asexual ones in their degree of specialization (Levene's test, *F*_1,27_ = 12.2, *p*-value < 0.002; electronic supplementary material, figure S1B).

To assess potential interactions between colour polymorphism and the number of different host plant species used, and thus study the contribution of predation to the realized feeding niches, we compared the degree of colour polymorphism within *Timema* species and populations with their degree of ecological specialization. At the species level, the size of the realized niche was correlated with the number of morphs of these species (correlation corrected with PGLS; *r* = 0.57, *p*-value < 0.003; electronic supplementary material, figure S1). Similar to the size of the species level realized niche, the asexuals contain two to five times fewer morphs than their sexual relatives, with the exception of *T. poppensis/T. douglasi*, in which both species have only a single morph (electronic supplementary material, table S1). By contrast, at the population level, we did not detect any link between colour polymorphism and the size of the realized feeding niche (Pearson's correlation; *r* = 0.14, *p*-value = 0.46; electronic supplementary material, figure S2B).

Survival and weight gain vary widely among the different studied *Timema* species when fed with different plants (*p* < 2.2 × 10^−16^ for survival and *F*_7,292_ = 8.94, *p* < 5.5 × 10^−10^ for weight gain; electronic supplementary material, figure S1A and figure 3*b*). We first tested whether sexual and asexual species feature different fundamental feeding niches by modelling, in each species pair, the survival and weight gain as functions of the species' reproductive mode and of the experimental feeding treatments (with interaction term). A significant interaction would indicate that sexual and asexual species have different fundamental feeding niches. We found a significant interaction for the pair *Timema californicum*/*Timema shepardi*; however, this was only the case for survival and only a trend for weight gain (table 1). We also found a significant interaction for the pair *T. poppensis*/*T. douglasi*, but only for weight gain, not survival (table 1). These results suggest that in at least two species pairs, asexuals and sexuals may have diverged in their fundamental niches.

**Table 1.** Effect of experimental feeding treatments and reproductive mode on survival and weight gain of insects.(***p < 0.001; *p < 0.05; ^•^*p* < 0.1.)

*Timema* species pair	factors tested in the statistical models	survival	weight gain
pair 2: *T. cristinae/T. monikensis*	[reproductive mode]	1.1 × 10^−05^***	*F*_(1,34)_ = 3.9, *p* = 0.054•
	[feeding treatment]	2.9 × 10^−09^***	*F*_(5,34)_ = 14.8, *p* = 10.0 × 10^−08^***
	[reproductive mode: feeding treatment] interaction	0.59	*F*_(2,34)_ = 3.9, *p* = 0.222
pair 3: *T. poppensis/T. douglasi*	[reproductive mode]	0.33	*F*_(1,107)_ = 4.9, *p* = 0.03*
	[feeding treatment]	0.20	*F*_(6,107)_ = 13.1, *p* = 4.6 × 10^−11^***
	[reproductive mode: feeding treatment] interaction	0.44	*F*_(6,107)_ = 5.5, *p* = 5.4 × 10^−05^***
pair 4: *T. californicum/T. shepardi*	[reproductive mode]	0.009***	*F*_(1,71)_ = 13.7, *p* = 0.0004***
	[feeding treatment]	4.8 × 10^−05^***	*F*_(6,71)_ = 19.4, *p* = 2.9 × 10^−13^***
	[reproductive mode: feeding treatment] interaction	0.0009***	*F*_(6,71)_ = 1.9, *p* = 0.09•
pair 5: *T. podura/T. genevievae*	[reproductive mode]	0.0004***	*F*_(1,80)_ = 4.4, *p* = 0.04*
	[feeding treatment]	6.4 × 10^−19^***	*F*_(6,80)_ = 22.1, *p* = 3.5 × 10^−15^***
	[reproductive mode: feeding treatment] interaction	0.35	*F*_(5,80)_ = 2.1, *p* = 0.08•

We then used the Tau index to test whether the breadth of the sexual and asexual species’ fundamental feeding niches also differ. Tau indices based on survival or weight gain were strongly correlated (Pearson's correlation, *r* = 0.96, *p* < 0.0001; figure 3*b*). We found significant differences in the fundamental niche breadths of sexuals compared to asexual species in two species pairs (*Timema cristinae*/*Timema monikensis* and *Timema podura*/*Timema genevievae*; electronic supplementary material, figure S1A and figure 3*b*). The remaining two pairs (*T. poppensis*/*T. douglasi* and *T. californicum*/*T. shepardi*) showed no significant difference (figure 3). Interestingly, *T. monikensis* and *T. genevievae*, which represent the most recent asexual lineage and oldest asexual lineage tested, respectively, were characterized by an opposite result. *Timema monikensis* was significantly more specialist (Tau based on weight gain = 0.27, 95% CI 0.22–0.29; survival = 0.21, 95% confidence interval (CI) 0.13–0.29) than its sexual relative *T. cristinae* (Tau based on weight gain = 0.47, 95% CI 0.41–0.55; survival = 0.46, 95% CI 0.34–0.58; figure 3*b*). On the contrary, the ancient asexual *T. genevievae* was significantly more generalist (Tau based on weight gain = 0.77, 95% CI 0.71–0.82; survival = 0.78, 95% CI 0.68–0.88) than its sexual sister species *T. podura* (Tau based on weight gain = 0.54, 95% CI 0.48–0.58; survival = 0.37, 95% CI 0.27–0.47; figure 3*b*). Finally, we found that the fundamental feeding niche breadths were not correlated with the sizes of their realized feeding niche, neither at the species level (Pearson's correlation; *r* = 0.13, *p* = 0.77; electronic supplementary material, figure S1A), nor at the population level (*r* = −0.14, *p* = 0.50; electronic supplementary material, figure S1B).

## 4. Discussion

We investigated if sexual and asexual *Timema* species differ in their *realized* feeding niches, i.e. the plant species they use as hosts under natural conditions, and how such differences come about. We find that asexual species generally feature smaller realized feeding niches than their sexual counterparts. Specifically, in four out of five sexual–asexual *Timema* species pairs, sexuals use about twice as many plants as asexuals in nature. In the fifth species pair, *T. poppensis*/*T. douglasi,* sexuals and asexuals use the same number of host plants. This species pair is probably an exception to the general pattern in *Timema* because of their ability to use the host plant redwood. We have shown in a previous study that sexual *Timema* species adapted to this specific host plant are ecologically highly specialized, perhaps because of reduced biotic pressures such as predation, parasitism and competition, on redwood [23]. This high level of ecological specialization in the sexual species *T. poppensis* makes a further specialization in the related asexual relatively unlikely.

To develop insights into how the narrower realized niches of asexual versus sexual *Timema* species come about, we quantified the size of their *fundamental* feeding niches, i.e. the range of plants *Timema* are able to use in the absence of the biotic pressures they normally face in nature. This allowed us to test if the size of the fundamental niche constrains the size of the realized niche, i.e. whether the reduced realized niche size in asexuals results from a reduced intrinsic ability to use different host plants. Fundamental feeding niche sizes varied significantly among all *Timema* species; however, there was no overall difference between reproductive modes. Fundamental niche sizes therefore do not explain why sexuals have broader realized niches than asexuals in *Timema*. Specifically, in two species pairs, the estimated fundamental niche size was very similar for sexuals and asexuals. In the other two pairs, the fundamental niche differed between sexuals and asexuals, however in opposite directions; in one species pair (*T. cristinae*/*T. monikensis*), the asexual species had a narrower fundamental niche than the sexual one, while in the other (*T. podura/T. genevievae*) the asexual species had a broader fundamental niche than the sexual one. The latter case is particularly interesting because *T. genevievae* is a very old asexual lineage (approx. 1.5–2 myr) and the oldest asexual *Timema* known (Schwander *et al.* [19]). The broad fundamental feeding niche in *T. genevievae* is consistent with predictions from the GPG theory, which posits that clones with broad environmental tolerances (i.e. broad fundamental niches) should be selectively favoured as such clones would be characterized by low variance in fitness across environments ([12]; figure 1*b*)*.* General-purpose genotypes are also believed to contribute to the persistence of one of the oldest known asexual species, the darwinulid ostracod *Darwinula stevensoni*. This species has probably existed as an obligate asexual for at least 25 million years and shows almost no morphological [36] or genetic [37] variability, yet it is a very common and cosmopolitan species [38] with broad tolerances for salinity and temperature [39].

In contrast to the old asexual *T. genevievae*, our findings in the youngest studied *Timema* asexual, *T. monikensis,* are consistent with the FNV model. This model suggests that the phenotypic distribution (i.e. fundamental niche) of a young, recently derived asexual lineage will be narrower than that of its genetically variable sexual ancestor ([8]; figure 1*a*). Indeed, *T. monikensis* is the only studied asexual that features a narrower fundamental niche than its sexual relative *T. cristinae* (figure 3*b* and electronic supplementary material, figure S1A).

Given that asexual *Timema* do not generally have narrower fundamental niches than sexual *Timema*, the narrow realized niches in asexuals are probably a consequence of biotic interactions that affect realized niche size in asexuals more strongly than in sexuals. A likely biotic factor affecting realized niches in *Timema* is selection imposed by predators (e.g. [24,25,28,40]. Several *Timema* species feature a natural colour polymorphism conferring crypsis on different host plants [24] and we therefore tested for links between colour polymorphism, realized niche size and reproductive mode in *Timema*. The sister species *T. douglasi* and *T. poppensis* do not feature any colour polymorphism, but in the four remaining species pairs, intra-population colour polymorphism is always higher in the sexual than asexual species. However, the level of polymorphism was only correlated to the size of the realized niche at the species level, not at the population level. Nevertheless, this higher degree of colour polymorphism in sexuals may allow for reduced predation rates on a larger number of plants relative to asexuals, potentially explaining the narrower realized niche size in asexual species.

In conclusion, we provide, to our knowledge, the first comparative study of realized and fundamental niches in replicated asexual–sexual species pairs. We found that sexual *Timema* species have a larger realized niche than asexual ones, but this difference is not explained by a similar difference in fundamental niche size. Thus, the smaller realized niches in asexuals are probably a consequence of biotic interactions that constrain asexuals more strongly than sexuals. Verifying potential links between population level colour polymorphism, realized feeding niche size and biotic interactions (especially predation and competition) will be a challenge for future studies. Finally, our finding that the oldest asexual *Timema* lineage is more generalist than its sexual relative could help explain its unusually long maintenance in the absence of sex.

## Supplementary Material

Tables S1 - S3, figures S1 and S2

## Data accessibility

All raw data is available from the Dryad Digital Repository at: http://dx.doi.org/10.5061/dryad.4j1h61t [41].

## References

[1] Neiman M, Meirmans PG, Schwander T, Meirmans S 2018 Sex in the wild: why field-based studies play a critical role in resolving the problem of sex. Evolution 72, 1194–1203. 29645091 10.1111/evo.13485

[2] Meirmans S, Meirmans PG, Kirkendall LR 2012 The costs of sex: facing real-world complexities. Q. Rev. Biol. 87, 19–40. (10.1086/663945) 22518931

[3] Case TJ, Taper ML 1986 On the coexistence and coevolution of asexual and sexual competitors. Evolution 40, 366–387. (10.1111/j.1558-5646.1986.tb00478.x) 28556058

[4] Halkett F, Kindlmann P, Plantegenest M, Sunnucks P, Simon JC 2006 Temporal differentiation and spatial coexistence of sexual and facultative asexual lineages of an aphid species at mating sites. J. Evol. Biol. 19, 809–815. (10.1111/j.1420-9101.2005.01055.x) 16674577

[5] Lehto MP, Haag CR 2010 Ecological differentiation between coexisting sexual and asexual strains of *Daphnia pulex*. J. Anim. Ecol. 79, 1241–1250. (10.1111/j.1365-2656.2010.01726.x) 20633199

[6] Bell G 1982 The masterpiece of nature: the evolution and genetics of sexuality. Cambridge, UK: CUP Archive.

[7] Doncaster CP, Pound GE, Cox SJ 2000 The ecological cost of sex. Nature 404, 281–285. (10.1038/35005078) 10749210

[8] Vrijenhoek RC 1984 Ecological differentiation among clones: the frozen niche variation model. In Population biology and evolution (eds Wohrmann K, Loeschcke V), pp. 217–231. Berlin, Germany: Springer.

[9] Case TJ 1990 Pattern of coexistence in sexual and asexual species of *Cnemidophorus* lizards. Oecologia 83, 220–227. (10.1007/BF00317756) 22160115

[10] Weeks SC 1993 The effects of recurrent clonal formation on clonal invasion patterns and sexual persistence: a Monte Carlo simulation of the frozen niche-variation model. Am. Nat. 141, 409–427. (10.1086/285481) 19426014

[11] Vrijenhoek RC, Parker ED Jr 2009 Geographical parthenogenesis: general purpose genotypes and frozen niche variation. In Lost sex. The evolutionary biology of parthenogenesis (eds Schon I, Martens K, Dijk P), pp. 99–131. Berlin, Germany: Springer.

[12] Lynch M 1984 Destabilizing hybridization, general-purpose genotypes and geographic parthenogenesis. Q. Rev. Biol. 59, 257–290. (10.1086/413902)

[13] Baker HG 1965 Characteristics and mode of origin of weeds. In The genetics of colonizing species (eds Baker HG, Stebbins GL), pp. 147–172. New York, NY: Academic Press.

[14] Parker ED, Selander RK, Hudson RD, Lester LJ 1977 Genetic diversity in colonizing parthenogenetic cockroaches. Evolution 31, 836–842. (10.1111/j.1558-5646.1977.tb01076.x) 28563714

[15] Hill WG, Robertson A 1966 The effect of linkage on limits to artificial selection. Genet. Res. (Camb.) 8, 269–294. (10.1017/S0016672300010156) 5980116

[16] Kondrashov AS 1988 Deleterious mutations and the evolution of sexual reproduction. Nature 336, 435–440. (10.1038/336435a0) 3057385

[17] Barton NH, Charlesworth B 1998 Why sex and recombination? Science 281, 1986–1990. (10.1126/science.281.5385.1986) 9748151

[18] Otto SP, Lenormand T 2002 Resolving the paradox of sex and recombination. Nat. Rev. Genet. 3, 252 (10.1038/nrg761) 11967550

[19] Schwander T, Henry L, Crespi BJ 2011 Molecular evidence for ancient asexuality in *Timema* stick insects. Curr. Biol. 21, 1129–1134. (10.1016/j.cub.2011.05.026) 21683598

[20] Riesch R et al 2017 Transitions between phases of genomic differentiation during stick-insect speciation. Nat. Ecol. Evol. 1, 1–13. (10.1038/s41559-017-0082) 28812654

[21] Bast J, Parker DJ, Dumas Z, Jalvingh K, Tran Van P, Jaron K, Figuet E, Galtier N, Schwander T 2018 Consequences of asexuality in natural populations: insights from stick insects. Mol. Biol. Evol. 35, 1668–1677. (10.1093/molbev/msy058) 29659991 PMC5995167

[22] Law JH, Crespi BJ 2002 Recent and ancient asexuality in *Timema* walking sticks. Evolution 56, 1711–1717. (10.1111/j.0014-3820.2002.tb01484.x) 12353765

[23] Larose C, Rasmann S, Schwander T 2018 Evolutionary dynamics of specialization in herbivorous stick insects. bioXriv (10.1101/367706) 30569559

[24] Sandoval CP 1994 Differential visual predation on morphs of *Timema cristinae* (Phasmatodeae: Timemidae) and its consequences for host-range. Biol. J. Linn. Soc. 52, 341–356. (10.1111/j.1095-8312.1994.tb00996.x)

[25] Sandoval CP 1994 The effects of the relative geographic scales of gene flow and selection on morph frequencies in the walking-stick *Timema cristinae*. Evolution 48, 1866–1879. (10.1111/j.1558-5646.1994.tb02220.x) 28565164

[26] Nosil P 2007 Divergent host plant adaptation and reproductive isolation between ecotypes of *Timema cristinae* walking sticks. Am. Nat. 169, 151–162. (10.1086/510634) 17211800

[27] Sandoval CP, Nosil P 2005 Counteracting selective regimes and host preference evolution in ecotypes of two species of walking-sticks. Evolution 59, 2405–2413. (10.1111/j.0014-3820.2005.tb00950.x) 16396181

[28] Nosil P 2004 Reproductive isolation caused by visual predation against migrants between divergent environments. Proc. R. Soc. Lond. B 271, 1521–1528. (10.1098/rspb.2004.2751) PMC1691753 15306325

[29] Jaenike J 1990 Host specialization in phytophagous insects. Annu. Rev. Ecol. Syst. 21, 243–273. (10.1146/annurev.es.21.110190.001331)

[30] Yanai I et al. 2004 Genome-wide midrange transcription profiles reveal expression level relationships in human tissue specification. Bioinformatics 21, 650–659. (10.1093/bioinformatics/bti042) 15388519

[31] Sandoval CP 2000 Persistence of a walking-stick population (Phasmatoptera : Timematodea) after a wildfire. Southwest. Nat. 45, 123–127. (10.2307/3672452)

[32] Simpson EH 1949 Measurement of diversity. Nature 163, 688 (10.1038/163688a0)

[33] Paradis E, Claude J, Strimmer K 2004 APE: analyses of phylogenetics and evolution in R language. Bioinformatics 20, 289–290. (10.1093/bioinformatics/btg412) 14734327

[34] Pinheiro JDB, DebRoy S, Sarkar D 2009 nlme: linear and nonlinear mixed effects models. *R Packag. version 3*. 1–137. See https://CRAN.R-project.org/package=nlme.

[35] R Core Team. 2017 R: a language and environment for statistical computing. Vienna, Austria: R Foundation for Statistical Computing.

[36] Rossetti G, Martens K. 1998 Taxonomic revision of the Recent and Holocene representatives of the Family Darwinulidae (Crustacea, Ostracoda), with a description of three new genera. *Bull. Inst. R. des Sci. Nat.* Belgique. Meded. K. Belgisch Inst. voor Natuurwetenschappen 68, 55–110.

[37] Schön I, Butlin RK, Griffiths HI, Martens K, Schon I, Butlin RK, Griffiths HI, Martens K 1998 Slow molecular evolution in an ancient asexual ostracod. Proc. R. Soc. Lond. B 265, 235–242. (10.1098/rspb.1998.0287)

[38] Griffiths HI, Butlin RK 1994 *Darwinula stevensoni*: a brief review of the biology of a persistent parthenogen. In The evolutionary ecology of reproductive modes in non-marine ostracoda (eds Horne DJ, Martens K), pp. 27–36. London, UK: Greenwich University Press.

[39] Van Doninck K, Schön I, De Bruyn L, Martens K 2002 A general purpose genotype in an ancient asexual. Oecologia 132, 205–212. (10.1007/s00442-002-0939-z) 28547353

[40] Nosil P, Crespi BJ, Sandoval CP 2003 Reproductive isolation driven by the combined effects of ecological adaptation and reinforcement. Proc. R. Soc. Lond. B 270, 1911–1918. (10.1098/rspb.2003.2457) PMC1691465 14561304

[41] Larose C, Parker DJ, Schwander T 2018 Data from: Fundamental and realized feeding niche breadths of sexual and asexual stick insects *Dryad Digital Repository*. (10.5061/dryad.4j1h61t) PMC6283937 30487310

