# Supplementary material for: Fundamental and realized feeding niche breadths of sexual and asexual stick insects
Source: Proc Biol Sci. 2018 Nov 28;285(1892):20181805. doi: 10.1098/rspb.2018.1805 (PMC6283937; doi:10.1098/rspb.2018.1805)
Supplement: Tables S1 - S3, figures S1 and S2 [file rspb20181805supp1.docx]

**SUPPORTING INFORMATION**

| **Table S1. Color morphs of ten *Timema* species** | | | | | | | | | | | | | | |
| --- | --- | --- | --- | --- | --- | --- | --- | --- | --- | --- | --- | --- | --- | --- |
|  | **Color morphs^1^** | | | | | | | | | | | | | |
| ***Timema* species** | **b** | **bl** | **br** | **db** | **dg** | **g** | **gr** | **oc** | **ol** | **sg** | **sgp** | **r** | **y** | **w** |
| *T. bartmani* |  |  | x |  |  | x | x |  | x | x | x |  |  |  |
| *T. tahoe* |  |  | x |  |  | x |  |  |  | x | x |  |  |  |
| *T. cristinae* | x |  |  | x |  | x | x | x |  | x |  | x | x |  |
| *T. monikensis* | x |  |  | x |  | x |  |  |  |  |  |  | x |  |
| *T. poppensis* |  |  |  |  |  | x |  |  |  |  |  |  | (x) |  |
| *T. douglasi* |  |  |  |  |  | x |  |  |  |  |  |  | (x) |  |
| *T. californicum* | x | (x) |  |  |  | x | x |  |  |  |  | x | x | (x) |
| *T. shepardi* |  |  |  |  |  | x |  |  |  |  |  |  | (x) |  |
| *T. podura* | x |  | x | x | x | x |  |  | x |  |  |  |  |  |
| *T. genevievae* |  |  |  |  | x |  |  |  |  |  |  |  |  |  |
| ^1^b, beige; bl, blue ; br, brown, db, dark brown, dg, dark grey; g, green; gr, grey; oc, ochre; ol, olive; sg, striped green; sgp, striped green with pink head; r, red; y, yellow; w, white  A cross indicates that a specific morph has been observed in a given species. A cross in brackets indicates that the morph was observed at a frequency of less than 0.1% in a given species, all populations combined. | | | | | | | | | | | | | | |

**Table S2. *Timema* populations sampled.** Number of individuals refers to the total number of individuals sampled in these locations on different host plants. We only selected locations in which at least three plants from the known host plant set were present.

| ***Timema* species** | **Location name (GPS coordinates)** | **Number of individuals per host plant sampled^1^** | **Morph frequency^2^ per sampling location** |
| --- | --- | --- | --- |
| *T. bartmani* | YMCA  (34°09'48.8"N 116°54'22.6"W) | 0 oak, 0 pin, 350 wf | 17%br, 34%g, 44%gr,5%ol |
|  | Jenks lake  (34°09'55.1"N 116°52'56.4"W) | 1 ced, 12 pin, 65 wf | 8%br, 23%gr, 4%ol, 12%, 38%sg, 15%sgp |
| *T. tahoe* | Bliss  (38°58'31.9"N 120°05'58.6"W) | 0oak, 0pin, 72 wf | 5%br, 32%gr, 36%sg, 27%sgp, |
|  | Vista  (38°45'34.8"N 120°11'57.2"W) | 0oak, 0pin, 51wf | 23%gr, 56%sg, 21%sgp |
|  | SN  (39°03'33.0"N 119°56'40.0"W) | 0oak, 0pin, 26wf | 57%sg, 43%sgp |
| *T. cristinae* | Ojai1  (34°31'01.7"N 119°16'39.7"W) | 245 lil, 73 mah, 6 mz, 70 oak, 5 toy | 2%b, 4%db, 87%g, 5%r, 1%y |
|  | Ojai2  (34°30'20.0"N 119°16'47.5"W) | 23lil, 62 mah, 11 mz, 28 oak | 3%b, 3%db, 69%g, 25%r |
|  | Ojai3  (34°31'59.6"N 119°14'51.8"W) | 8 cha, 2 lil, 20 mah, 8 oak | 9%b, 6%db, 59%g, 6%gr, 4%r, 9%sg, 7%y |
|  | WTA1  (34°30'46.6"N 119°46'41.7"W) | 597 cha, 317 mah, 78 oak | 3%b, 10%db, 23%g, 3%oc, 61%sg <1%y |
|  | WTA2  (34°30'22.3"N 119°46'05.3"W) | 81 cha, 1 mah, 8 mz, 9 oak, 2 toy | 3%b, 10%dgb 21%g, 2%oc, 64%sg |
|  | WTA3  (34°30'56.8"N 119°46'43.7"W) | 60 cha, 24 lil, 5 mz, 7 toy | 2%db, 21%g, 7%oc, 69%sg, 1%r |
|  | WTA4 | 100 cha, 253 mah, 2 oak | 4%b, 7%db, 80%g, 2%oc, 7%r |
|  | (34°29'58.3"N 119°43'08.2"W) |  |  |
| *T. monikensis* | Sycamore  (34°06'33.7"N 118°54'51.0"W) | 0 cha, 9 lil, 0 oak, 0 rdw | <1%b, 5%db, 95%g |
|  | For Sale  (34°06'53.6"N 118°51'11.3"W) | 0 lil, 0 oak,13rdw | 9%b, 13%db, 69%g, 9%y |
|  | Decker  (34°06'10.6"N 118°51'42.4"W) | 12 lil, 0 mz, 0 oak, 0 rdw | 23%b, 35%db, 42%g |
| *T. poppensis* | Fish Rock  (38°49'05.1"N 123°35'03.5"W) | 137 df, 0 lil, 14 rdw | 100%g |
|  | Fish Rock2  (38°54'57.1"N 123°18'00.6"W) | 34 df, 0 oak, 32 rdw | 100%g |
|  | Bear Creek  (37°09'56.2"N 122°00'56.4"W) | 85 df, 0 oak, 35 rdw | 100%g |
|  | Madonna  (37°01'07.5"N 121°43'32.0"W) | 0 mz, 0 oak, 403 rdw | 100%g |
| *T. douglasi* | Orr Springs 1  (39°12'44.5"N 123°18'30.2"W) | 42 df, 0 cha, 2 mz, 0 oak | 99.99%g, 0.01%y |
|  | Manchester 12  (38°58'57.2"N 123°28'10.4"W) | 1073 df, 5 mz, 0 oak | 100%g |
| *T. californicum* | Skyline  (37°14'43.6"N 122°06'37.0"W) | 2 cha, 18 mz, 43 oak | 8%b, 89%g, 3%r |
|  | Saratoga  (37°11'47.0"N 122°02'27.1"W) | 4 cha, 12 mz, 4 oak | 12%b, 82%g, <1%gr, 4%r, 1%y |
|  | Summit  (37°02'43.2"N 121°45'11.6"W) | 51 mz, 0 oak, 0 rdw | 10%b, 2%bl, 85%g, 3%r |
| *T. shepardi* | Elk  (39°16'42.2"N 122°55'39.6"W) | 0 lil, 304 mz, 0 pin, 0 oak | 100%g |
|  | Manchester 2  (38°57'22.4"N 123°32'04.9"W) | 0 df, 30 mz, 0 oak | 100%g |
|  | Orr Springs 2  (39°12'02.2"N 123°17'38.1"W) | 1df, 0 lil, 200 mz | 99.99%g, 0.01%y |
| *T. podura* | Indian  (33°47'50.5"N 116°46'35.5"W) | 79 cha, 60 lil, 0 mah, 7 mz, 0 oak | 5%b, 36%db, 24%dg, 23%g, 2%oc, 10%ol |
|  | Poppet  (33°51'36.9"N 116°50'20.4"W) | 45 cha, 0 lil, 0 mz | 4%b, 14%db, 14%dg, 45%g, 23%ol |
| *T. genevievae* | HW20  (38°59'38.4"N 122°31'26.4"W) | 60 cha, 0 mz, 0 oak | 100%dg |
|  | Antonio  (37°19'42.0"N 121°29'07.6"W) | 248 cha, 0 mah, 0 oak | 100%dg |
| **^1^**Plant name abbreviations: ced, insense cedar (*Calocedrus decurrens*); cha, chamise (*Adenostoma fasciculatum*); df, douglas fir (P*seudotsuga menziesii*); lil, califonian lilac (*Ceanothus spp*); mah, montain mahogany (*Cercocarpus betuloides*); mz, manzanita (*Arctostaphylos spp*); oak, oak (*Quercus spp*); pin, pinus (*Pinus spp*); rdw, redwood (*sequoia sempervirens*); toy, toyon (*Heteromeles arbutifolia*).  ^2^For morph name abbreviation, see Table S1 | | | |

**Table S3. Overview of the *Timema* ssp used for the study of the fundamental feeding niche**

| ***Timema* species** | **Reproductive mode** | **Original host plant** | **GPS coordinates** | **Number of individuals^1^** |
| --- | --- | --- | --- | --- |
| *T. cristinae* | Sexual | *Ceanothus thyrsiflorus* | 34°30’19.7’’N 119°16’53.6’’W | 70 |
| *T. monikensis* | Asexual | *Cercocarpus betuloides* | 34°06’53.7’’N 118°51’09.7’’W | 100 |
| *T. poppensis* | Sexual | *Pseudotsuga menziesii* | 37°09’56.7’’N 122°00’55.0’’W | 70 |
| *T. douglasi* | Asexual | *Pseudotsuga menziesii* | 38°58’57.2’’N 123°28’10.4’’W | 70 |
| *T. californicum* | Sexual | *Arctostaphylos glauca* | 37°20’41.3’’N 121°37’59.6’’W | 80 |
| *T. shepardi* | Asexual | *Arctostaphylos glauca* | 39°12’02.8’’N 123°17’38.2’’W | 70 |
| *T. podura* | Sexual | *Adenostoma fasciculatum* | 33°41’12.3’’N 116°42’11.2’’W | 105 |
| *T. genevievae* | Asexual | *Adenostoma fasciculatum* | 37°19’42.0’’N 121°29’07.6’’W | 70 |

**^1^** number of individuals used in the feeding experiment


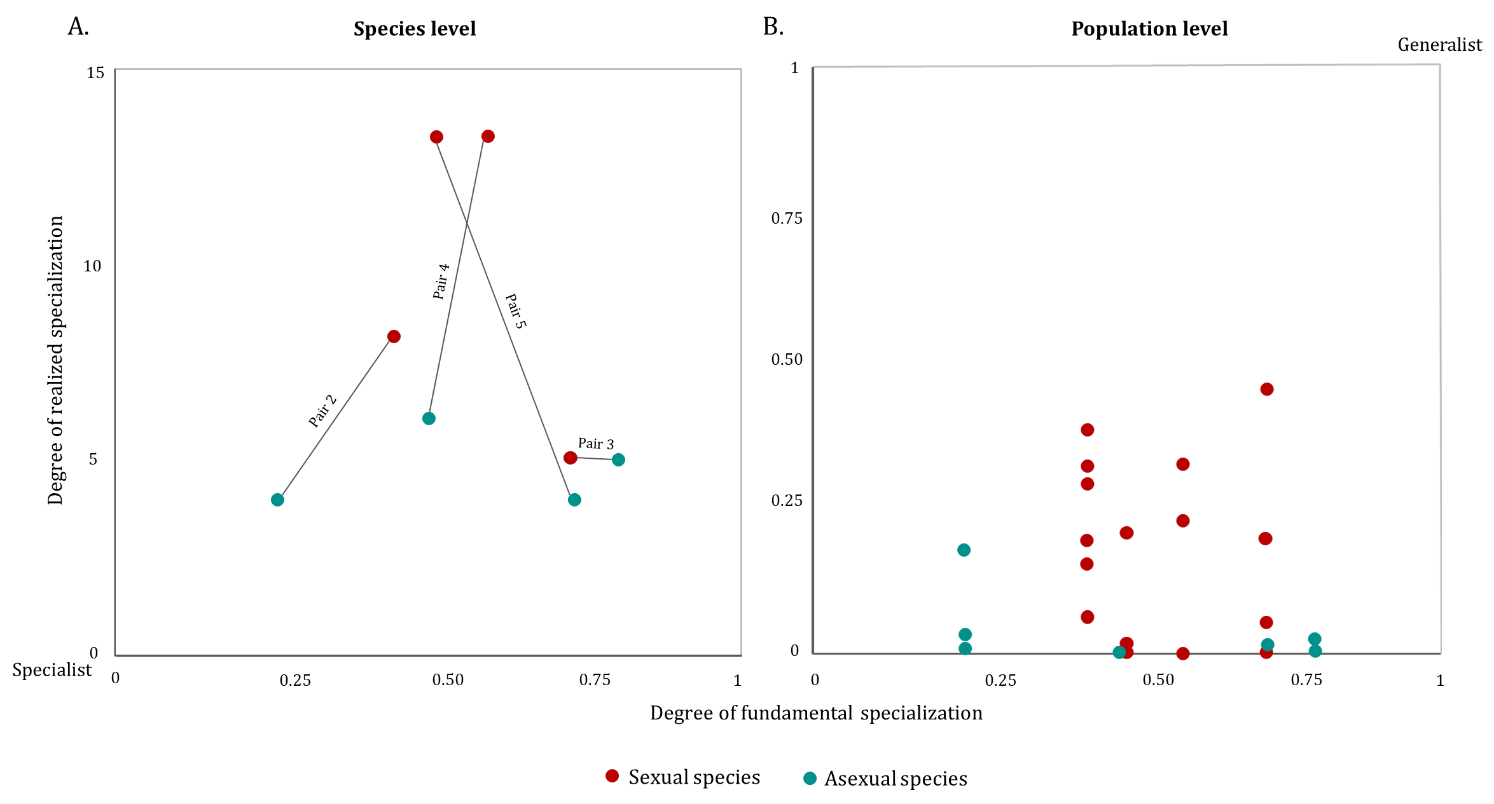


**Figure S1. Realized and fundamental feeding niche breadths of sexual and asexual stick insects are not correlated.** Shown is the specificity index Tau (calculated from the weight gain of insects) as a function of the realized feeding niche at the species level **(A)** or at the population level **(B)**. For species pair numbers, see Fig. 2 in the main text.


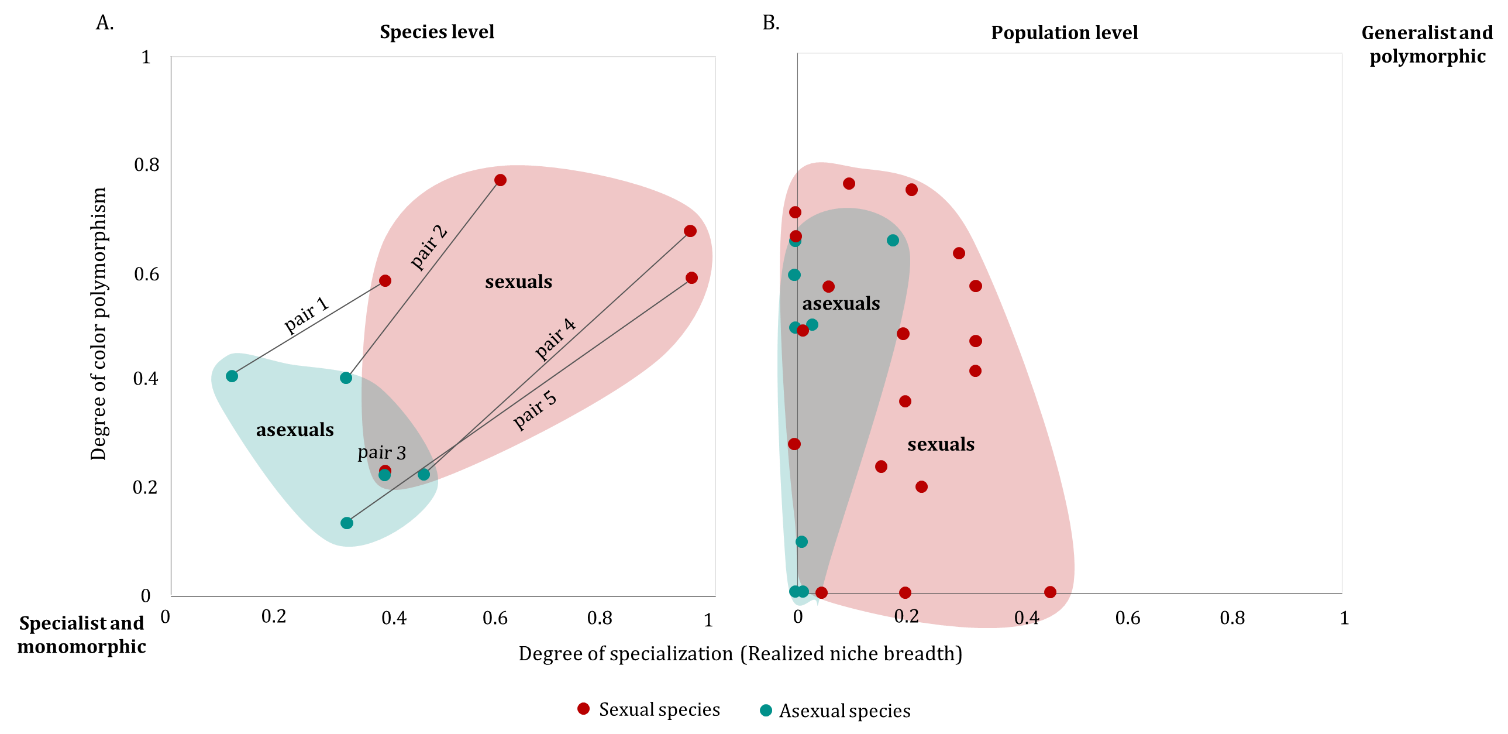


**Figure S2. Correlation between color polymorphism and realized feeding niche breadth of *Timema* at the species (A) and at the population (B) levels**. At the species level (A), the polymorphism levels and realized feeding niche sizes are estimated from a count of the different color morphs and of the known host plants in each species respectively. At the population level (B), the polymorphism level is estimated using the inverse Simpson diversity index, and the realized feeding niche size is estimated using the Tau index. In this case, 0 corresponds to specialism and monomorphism, and 1 corresponds to generalism and extreme polymorphism.
